# Isothermal Amplification of Nucleic Acids Coupled with Nanotechnology and Microfluidic Platforms for Detecting Antimicrobial Drug Resistance and Beyond

**DOI:** 10.34172/apb.2022.004

**Published:** 2021-01-30

**Authors:** Seyedeh Zahra Alamolhoda, Nosratollah Zarghami, Houman Kahroba, Ahmad Mehdipour, Mohammad Pourhassan-Moghaddam, Rana Jahanban-Esfahlan, Morteza Milani

**Affiliations:** ^1^Department of Medical Biotechnology, School of Advanced Medical Sciences, Tabriz University of Medical Sciences, Tabriz, Iran.; ^2^Student Research Committee, Tabriz University of Medical Sciences, Tabriz, Iran.; ^3^Department of Molecular Medicine, School of Advanced Medical Sciences, Tabriz University of Medical Sciences, Tabriz, Iran.; ^4^Department of Tissue Engineering, School of Advanced Medical Sciences, Tabriz University of Medical Sciences, Tabriz, Iran.; ^5^Infectious and Tropical Diseases Research Center, Tabriz University of Medical Science, Tabriz, Iran.

**Keywords:** Biosensing, Isothermal amplification techniques, Antibiotic drug resistance, Nanotechnology, Microfluidics

## Abstract

Antibiotic resistance is one of the serious health-threatening issues globally, the control of which is indispensable for rapid diagnosis and treatment because of the high prevalence and risks of pathogenicity. Traditional and molecular techniques are relatively expensive, complex, and non-portable, requiring facilities, trained personnel, and high-tech laboratories. Widespread and timely-detection is vital to the better crisis management of rapidly spreading infective diseases, especially in low-tech regions and resource-limited settings. Hence, the need for inexpensive, fast, simple, mobile, and accessible point-of-care (POC) diagnostics is highly demanding. Among different biosensing methods, the isothermal amplification of nucleic acids is favorite due to their simplicity, high sensitivity/specificity, rapidity, and portability, all because they require a constant temperature to work. Isothermal amplification methods are utilized for detecting various targets, including DNA, RNA, cells, proteins, small molecules, ions, and viruses. In this paper, we discuss various platforms, applications, and potentials of isothermal amplification techniques for biosensing of antimicrobial resistance. We also evaluate the potential of these methods, coupled with the novel and rapidly-evolving platforms offered by nanotechnology and microfluidic devices.

## Introduction

### Significance of antimicrobial resistance

Antimicrobial resistance (AMR), as a highly concerning issue all over the world, affected the health programs in countries. The significance of AMR makes the United States, National Institute of Health,^1^ World Health Organization (WHO),^2^ Centers for Disease Control and Prevention (CDC), and the United Nations (UN) to deal with this issue. The increasing rate of AMR and the emergence of new pathogens from bacteria to viruses, as the coronavirus disease 2019 (COVID-19) pandemic, is a major challenge.^3-5^

Extensive growth in antibiotic resistance incidence is confirmed by WHO’s new Global Antimicrobial Surveillance System (GLASS) studies by evaluating 500 000 suspected cases with bacterial infection across 22 different countries. Antibiotic resistance to at least one of the common antibacterial agents was apparent among suspected cases for sepsis ranging from 0-82%. Resistance to penicillin as the common antibacterial agent for pneumonia treatment ranged from 0%-51%. Also, 8%-65% of all diagnosed *Escherichia coli*infections were resistant to ciprofloxacin as the standard medicine in treating urinary infection.^6^ Not to forget the development of resistance to *Mycobacterium tuberculosis*, the causative agent for tuberculosis (TB), followed by WHO since 1994. Not to mention that TB as a dangerous and life-threatening disease, affects about one-third of the world’s population.^7^

CDC Threats Report for AMR in 2019, indicatesthat unsuccessfully-treated bacterial infections due to AMR, which claims at least 700 000 lives per year around the world, is anticipated to reach more than 10 million deaths per year up to 2050, with a burden of US $100 trillion to the global economy by loss of productivity. Only in the USA, more than 2.8 million multidrug-resistant bacterial infections are reported per year, which are responsible for about 35 000 deaths and $20 billion health-care expenditures.^8^ Unlike many dedicated efforts to prevent and control infection in the US to reduce the mortality rate, the number of people facing antibiotic resistance is still too high. Moreover, in 2020, WHO ranked AMR as one of the top 10 global public health threats facing humanity, requiring urgent multi-sectoral action to achieve the Sustainable Development Goals (SDGs). AMR’s economic cost is significant, along with death and disability, prolonged hospitalization, and demands on high-cost medicines; also, the financial challenges for those impacted patients is a critical point. Low- or unavailability of effective antimicrobial strategies and agents limit modern medicine’s success rate in treating infections mainly during major surgeries and cancer chemotherapy.^9-12^ The emergence and spread of new forms of resistance remain a concern. What’s even more alarming is the con-infection of the unresolved, worldwide infections such as TB with other emerging infections, such as COVID-19. Reports from five different countries indicated that about 6.9% of the patients with COVID-19 have bacterial infections (3.5% diagnosed concurrently and 14.3% post-COVID-19); also this percentage is higher in patients who require intensive critical care. Nevertheless, a US multicenter study revealed that about 72% of the COVID-19 patients are administered antibiotics even with no clinical symptoms, promoting the AMR in bacteria. AMR can be deteriorated under the influence of COVID-19 infection due to the overprescription of antibiotics, continuous misapplication in agriculture, and the dearth of antimicrobials in the development pipeline. Competing global priorities are decreasing the AMR eradication activities, such as measures for multidrug-resistant tuberculosis.^13,14^

### Polymerase chain reaction (PCR) techniques for detection of AMR

The introduction of PCR was a big evolution for researchers and clinicians in biological and clinical research, which opened a bride gate to a new molecular and biological research and diagnostics world.^15^ Regarding its potential for detecting AMR, using a rapid high-throughput PCR, one study reported an evaluation of more than 7500 highly antibiotic-resistant clinical isolates of *Pseudomonas aeruginosa, E. coli*,* Proteus mirabilis,*and *Klebsiella pneumonia* for antibiotic-resistance genes. These isolates were obtained from different hospitals including Europe, Asia, South America, North America, Africa, and Oceania. Authors compared genetic data with phenotypic resistance across cephalosporins, penicillins, aminoglycosides, carbapenems, fluoroquinolones, trimethoprim-sulfamethoxazole, and macrolides. Using PCR, average positive predictive values for genotypic prediction of phenotypic resistance were 92% for *P. aeruginosa*, 87% for *P. mirabilis*, 93% for *K. pneumonia*, 91% for *E. coli*.^16^

Early identification of genes associated with a specific antibiotic resistance mechanism using a PCR-based approach can reduce unnecessary antibiotic consumption by predicting a gain of bacteria resistance within hours.^17^ PCR also can impact hospital-based transmission of antibiotic-resistant organisms by allowing for timely implementation of appropriate precautions when an organism is predicted to have a resistance gene.^18^ Equally, reverse transcriptase-PCR (RT-PCR) is the number one diagnostic test for detecting COVID-19 across the world.^19-22^

Although PCR technology may impact patient care, further studies are required to assess its utility in the patient care setting. The cost-benefit of PCR as the premier method for AMR detection needs to be reevaluated in the account of the facility needs, resources, costs, and the prevalence of resistant isolates, and the need for adequate and trained personal,^23^ especially in the resource-limited settings/regions.^3,24^

### Isothermal amplification methods for the detection of AMR

Since the early 1990s, isothermal nucleic acid amplification tests have emerged as an essential diagnostic tool, with applications in clinical diagnosis, environmental monitoring, and testing food quality.^25^ These methods included loop-mediated isothermal amplification (LAMP), nucleic acid sequence-based amplification (NASBA), helicase-dependent amplification (HDA), strand displacement amplification (SDA), rolling circle amplification (RCA), recombinase polymerase amplification (RPA), and transcription-mediated amplification (TMA).^26^ Amplification of nucleic acids using a constant temperature (isothermal) eliminated the need for different temperature cycles, favored enzyme activity, reduced sample consumption, and saved time to produce fast results. Equally, the fully closed and safe micro-structured device can reduce contamination risk.^27^ Together, isothermal amplification-based tests are user-friendly, simple, rapid, sensitive, portable, and require less energy than PCR/RT-PCR and can address the need for a simple, quick, available, and culture-free molecular diagnostic method for widespread assessing of AMR.^28,29^ To further simplify these methods, some of them analyze the sample’s raw lysates without the need for nucleic acid purification.^30^

Besides early pathogen screening and antibacterial resistance identification, isothermal amplification methods are also applied for cancer cell detection. Recent studies are focused on cancer cell detection using isothermal amplification methods, particularly via identifying microRNAs involved in cancer progression, invasion, and metastasis.^31,32^ Further, integration of isothermal amplification methods with newly developed microfluidics and nanotechnology-based approaches generated novel tools for the identification of cancer cells and AMR.^33-35^

This review aims to discuss the use of different isothermal-based systems for nucleic acid amplification and highlight their potential in the context of detecting AMR. We also discuss the integration of isothermal-based systems with nanotechnology and microfluidics and evaluate their potential to realize miniature yet sensitive biosensing platforms for the screening of antibiotic drug resistance.

## Isothermal amplification platforms for detecting AMR

In this section, we review the variety and potential of isothermal-based-biosensing platforms to detect human-related microbial pathogens. As the sensitivity and specificity of these methods vary among different types of the samples and analytical method used, these are discussed within the text.

### Recombinase polymerase amplification

**Figure 1:**
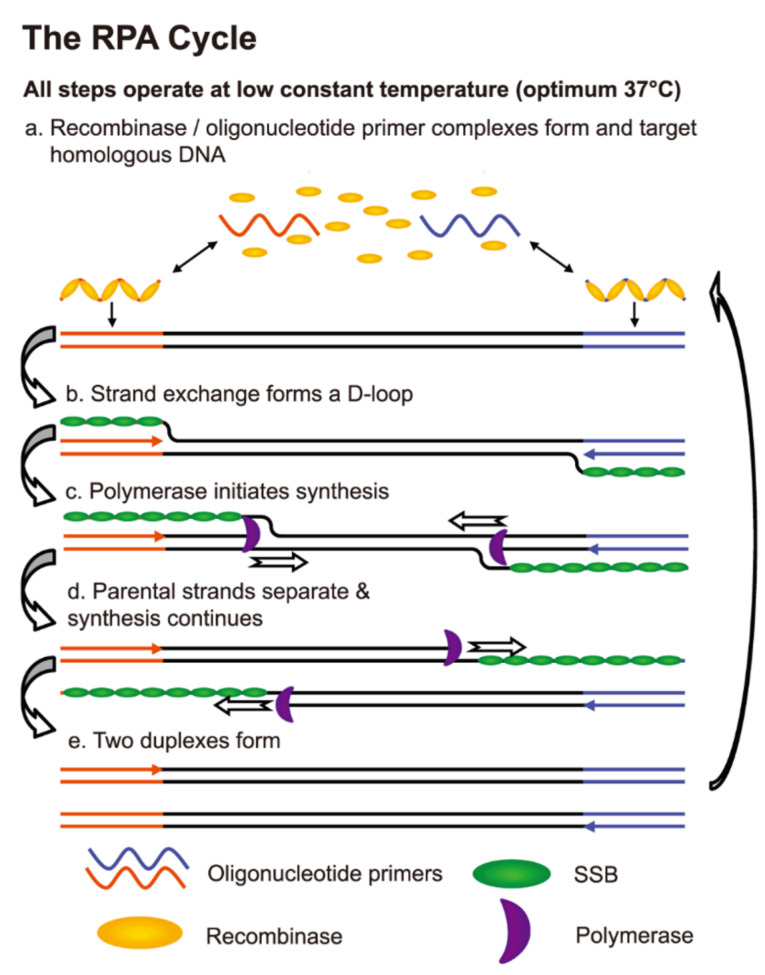
An overview of RPA for rapid biosensing of TB. SSB: Single-strand binding protein. Adopted from Boyle et al.^42^

RPA is an isothermal method, which denatures the genomic target DNA using recombinase-primer complexes followed by stabilizing the single-stranded DNA (ssDNA) with the help of ssDNA binding proteins (Figure 1). The RPA detection is very similar to Taq-Man hydrolysis probes except that the probe includes tetrahydrofuran, a basic site analog and cleaved by endonuclease IV. The strand displacing Bsu compared with Taq is more resistant to chemical inhibition.^36^ Since the proteins are active elements in DNA denaturation, there is no need for high denaturation temperatures. Thus, the reaction occurs in temperatures ranging from 37-42°C and more rapid than the PCR method, usually 5-7 min.^36-38^ The excessive sensitivity of RPA allows the technique to detect tens of the target’s copies.^38-41^

Despite little use of RPA in clinical applications, there is evidence that this method can potentially detect bacterial, viral, and protozoan human pathogens. Moreover, RPA is capable of detecting blood fluke *Schistosoma japonicum*,^38^ *Giardia*, *Cryptosporidium* and *Entamoeba*,^41,43^ as well as the human immunodeficiency virus *(*HIV*)*,^44,45^ Chikungunya virus (CHIKV),^37^ Rift Valley fever virus,^46,47^ foot-and-mouth disease virus (FMDV),^48^ Middle East respiratory coronavirus,^49^ Crimean Congo hemorrhagic fever virus (CCHFV),^50^ and bovine coronavirus.^51^ The bacterial species detected by RPA include *M. tuberculosis*,^52,53^ *Neisseria gonorrhoeae*, methicillin-resistant* Staphylococcus aureus*(MRSA) and *Salmonella enterica*.^54^

*Pectobacterium* species are important contributors to soft rot in fruit and vegetable products such as tomatoes and potatoes.^55^ So one needs precise and simple methods for rapidly identifying the pathogen for timely management of injuries. The RPA, combined with a lateral flow device (LFD) was developed to detect *Pectobacterium*spp. directly from the contaminated plant material without the need for DNA isolation.^56^ The specificity of RPA-LFD was determined with 26 *Pectobacterium*spp. and 12 non-*Pectobacterium* species with no false positive or false negative outcomes. The sensitivity and accuracy of the test were determined by the development of RPA primers and probes. Both sensitivity and spiked sensitivity methods obtained the detection limit of 10 fg. When targets were clearly identified from infected potatoes and tomatoes, no inhibitory effect was observed on the RPA assay. Furthermore, this method can also be used to detect reservoir hosts for *Pectobacterium* species.^57^ Moreover, RPA-LFD is used for detection of the plant pathogen *Phytophthora sojae*, with extraordinary specificity (negative results against 24 other Phytophthora, 14 fungal species, and one *Globisporangium*), high sensitivity (10 pg/50-μL genomic DNA of *P. sojae*) within 5 min.^58^ Likewise, the RPA-LFD principle is applied for screening of African swine fever virus with the sensitivity of 150 copies per reaction within 10 min at 38°C, and positive rates (10/145) comparable to RT-PCR.^59^

RPA is recognized as a particular method in diagnostic and clinical use and resistant to false positives (type I errors). In many cases, a specificity of 100% is indicated.^37,38,40,45^ Due to the health risks of diagnostic and therapeutic errors, high specificity is an important feature in diagnostic analyzes. Type II errors (false negatives) may always occur if the pathogenic agent in the sample is low, but accurate RPA sensitivity reduces this error.^60^

In one study, Nelson et al^61^ developed a new test for RPA to identify the Macrolide Efflux A or mef (A) gene that encodes a pump responsible for causing bacterial resistance to 14- and 15-membered macrolide antibiotics (including erythromycin and azithromycin).^62,63^ This gene is also expressed by *S. pyogenes*, which is located on a transposon that is integrated into a pro-phage.^64,65^ This gene was initially identified in *S. pyogenes* and *S. pneumoniae* and has so far been identified on a range of Gram-positive and Gram-negative bacteria.^66^

### Multiple displacement amplification

**Figure 2:**
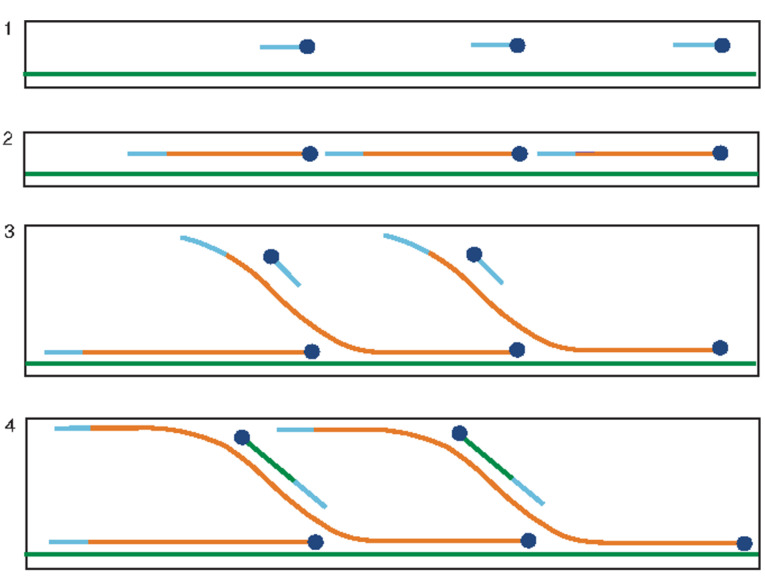
**Overview of the principle of MDA.** 1) Random hexamers (blue lines) bind to ssDNA (green lines). 2) DNA synthesis is formed by Phi29 enzyme (blue circles) to create newly synthesized double-stranded DNA (dsDNA) (orange lines). 3) Primers bind to the newly synthesized DNA, and Phi29 displaces the strands and continues the polymerization. 4) Triggering polymerization on new strands leads to the formation of a hyperbranched structure. dNTP: deoxy nucleotide three phosphates. Adapted from Spits^71^ with permission from Springer Nature, Copyright 2006.

Multiple displacement amplification (MDA) is another PCR-independent technique to amplify the amounts of DNA with a lower frequency of errors.^67^ The test starts by annealing the random hexamer primers to the template DNA followed by a high-fidelity DNA polymerase that works to continue the polymerization and amplification of the target DNA. Phi29 is the most used polymerase enzyme in this method. Since Phi29 is a high processivity enzyme, the MDA method can produce larger-sized products than PCR (greater than 70 kb),^68^ rendering it a superior candidate for WGS compared to WGS-PCR, which generates less DNA than 1000 bp long.^69^ Moreover, 3’ to 5́ proofreading activity of Phi29 enzyme decreases the error rate to 1 in 10^6^ to 10^7^ basis.^70^ The optimum temperature for performing the reaction is 30°C.^71^ (Figure 2)

MDA is an appropriate method for DNA amplification from small biological samples. This technique’s application is expanding due to particular advantages, including the capability to analyze defective DNA samples,^72^ production of long DNA fragments for the analyzing molecular markers, lack of sensitivity to GC content, and self-limitation of MDA reaction.^72-74^ The major application of this method relies on the diagnosis of TB. In this line, a study was performed to evaluate the new two-step PCR-MDA by combining the MDA method IS6110-specific PCR to detect TB infection. In this experiment, the MDA assay was used as an amplification step to increase the amount of DNA in clinical samples of saliva followed by IS6110 specific PCR. In this two-step assay, DNA polymerase, Phi29 was used to amplify the entire genome.^75^

### Helicase dependent amplification

**Figure 3:**
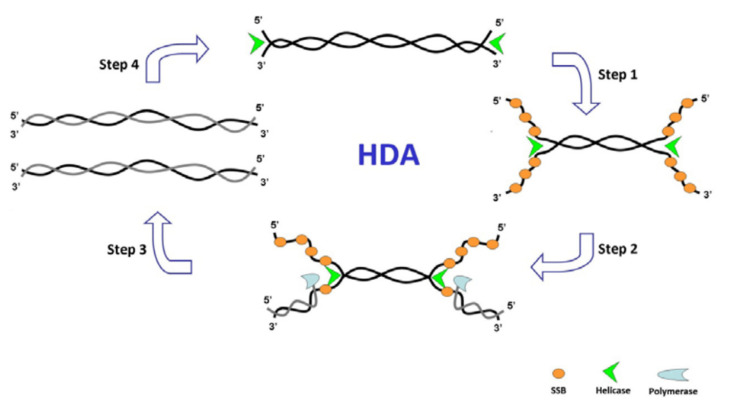
Schematics of helicase dependent amplification. SSB: single-strand binding proteins. Adopted from Zanoliet al.^30^

HDA is an isothermal method to amplify DNA fragments at lower temperatures compared with PCR and uses the helicase enzyme to denature the DNA double helix (Figure 3).^76,77^ HDA starts with the function of helicase enzyme separating DNA double helix and forming the ssDNA with DNA-binding proteins (ssBPs). Sequence-specific primers designed for the target DNA anneal to the template DNA’s borders during the third step. Then DNA polymerase extends the primers to generate new dsDNA. This reaction repeats for these new products.^78^

The application of HDA is widespread for the detection of genetic polymorphisms to microbial tracking. In earlier attempts, HDA was used for the detection of *Clostridium difficile* due to its simplicity.^79^ Later, it was used to rapidly detect *S. aureus* using a short DNA fragment specific to this microorganism.^80^ Recently, Kolm et al^81^ employed this method to detect the 16srRNA in *Phylum bacteroides* as water fecal pollution indicators. This marker is now known as “BacR” which was traditionally assessed by quantitative PCR (qPCR). The authors compared the newly designed HDA with the qPCR method and demonstrated that amplicon selection and primer design restricted the HDA performance. They also determined the limit of detection (LOD_95%_) for each technique and found that HDA performance was excellent compared to qPCR with 5.2-10.3 copies for HDA vs. 2.6-5.2 copies qPCR.

### Loop-mediated isothermal amplification

Among different isothermal methods, LAMP ranks the first, as 60.7% of all the publications listed in Web of Science by 2019 are reported to use this method, followed by RCA (16.2%). LAMP is reputed for its robust and high analytical sensitivity and specificity to amplify target DNA. This is because this method uses up to six primers. However, this also increases the risk of forming primer dimers due to many long primers.^82^ The isothermal and energy-efficient amplification requirements render LAMP as the first candidate for low-cost analysis and diagnostics at the point of need.^25^ Another advantage of LAMP is its ability to be combined with the RT enzyme to use RNA as a template.^83^ More importantly, LAMP assays are shown to excel RT-PCR performance with a ~100 times more sensitivity, capable of detecting viral RNA template down to 1–10 copies per reaction.^84^

Mechanistically, LAMP employs the strand displacement principle and uses two pairs of specific primers to detect six different template DNA areas. It is described for the first time by Notomi et al.^85^ In contrast to PCR reaction, DNA denaturation and primer annealing in LAMP occurs in a non-temperature-dependent manner, which relies on the kinetics of the reaction and the physical distance between the target sequence and primers in a loop.^86,87^

**Figure 4:**
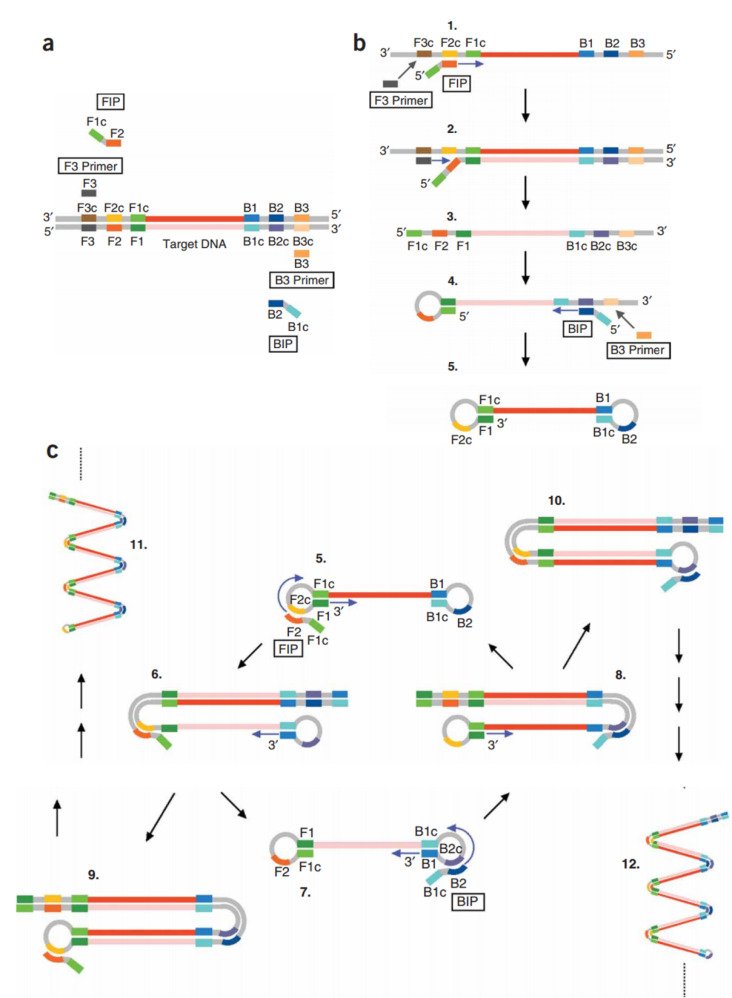
Schematic representation of loop-mediated amplification. Adapted from Tomitaet al^90^ with permission from Springer Nature Copyright 2008.

The LAMP technique uses Bst DNA polymerase with the ability to mediate strand displacement reactions. The process is generally divided into two stages: the beginning of the structure production and the cycling reinforcement phase. In the first stage, all four primers are used, while in the second stage, only two internal primers are used. Single-stranded DNA is released by DNA synthesis.^26^ The ssDNA released by an outer primer (F3) acts as a template for the next step of synthesis initiated by BIP and B3 and generates a stem-loop DNA structure.^88^ Starting with a complimentary inner loop primer on the product will continue the cycling amplification process of each inner primer intermittently. This process is seen in a geometric aggregation of 10^9^ copies of the target sequence in less than one hour.^89^ The end products of stem-loop DNA can be distinguished by multiple repeats and cauliflower-like structures with multiple loops even using the real-time method and the endpoint method.^83^ These steps are shown in detail in Figure 4. In this figure, (a) shows the primer design of the LAMP reaction. Six distinct regions are selected labeled F3, F2, F1, B1c, B2c, and B3 from the 5’end in which c represents complementary. The inner and outer primers used are FIP and BIP, and F3 and B3, respectively. (b) The process of synthesis starts from FIP. The F2 region binds to the F2c to initiate elongation. BIP similarly helps DNA amplification. The F3 binds to the F3c, and strand displacement DNA synthesis occurs. The elongated DNA from FIP is replaced and released. The resulted ss-DNA forms a loop in 5’ ends (structure 4).

DNA synthesis continues with the ss-DNA and BIP and B3 primers to generate structure 5 containing the loop structure at both ends (dumbbell-like structures). (c) Cycling amplification step. Structure 5 is used as a template, and self-primed DNA synthesis is started from the 3’ end of the F1 region and the annealing of FIP to single-stranded F2c region in the loop structure. After completion of the several steps, structure 7, complementary to structure 5, is generated. Structure 5 results from a similar reaction from structure eight, which originated from structures 5-7. Intermediate structures, including structures 7a and 9a, and structures 5a and 10a, are the products of structures 9 and 10, respectively. However, the displaced strands 7a and 5a are dumbbell-like structures 7 and 5, respectively. More elongated structures such as 11 and 12 are also generated.^90^

As mentioned before, because of the large number of primers, the LAMP’s specificity and sensitivity are greatly increased. Therefore, it is considered a useful molecular method for detecting pathogens, such as MRSA in blood cultures. Equally, WHO published policy guidance for the use of LAMP for the diagnosis of pulmonary tuberculosis.^91^ Because *S. aureus* is one of the most important pathogens that is causing deadly infections, its rapid and accurate diagnosis by the LAMP method has produced satisfying results.^92^ A recent study by Foo et al on *Entamoeba histolytica* DNA samples reported better LOD results for LAMP and its rapidity in nucleic acid amplification compared with PCR, nested PCR, and real-time PCR. This report, along with previously mentioned studies, confirmed the LAMP technique’s advantages over the previous complex molecular methods in the rapid and sensitive detection of pathogenic microorganisms.^93^

### Nucleic acid sequence-based amplification

**Figure 5:**
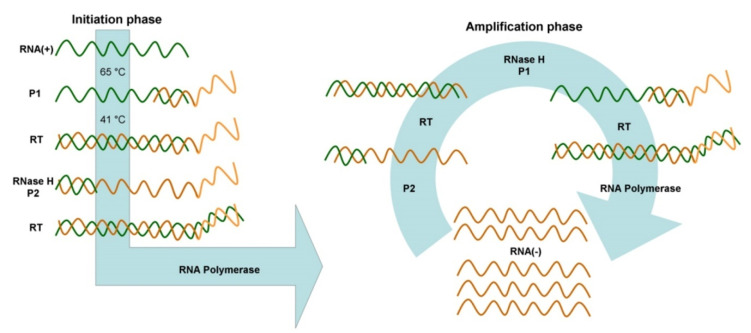
**Schematics of NASBA composed of two initiation and amplification cycles.** Adopted from Zanoli et al.^30^

NASBA^94^ that renowned as self-sustained sequence replication (3SR),^95^ is known as a primer-dependent method that can utilize the continuous proliferation of nucleic acids in a single mixture at a constant temperature. This method is comparable to RT-PCR in that it is used to amplify single-stranded RNAs (ssRNAs). This method is based on the function of three enzymes: reverse transcription, RNase H, and DNA-dependent RNA polymerase. First, reverse transcriptase (RT) and RNase H transcripts complementary DNA (cDNA) from RNA. Then DNA-dependent RNA polymerase produces antisense RNAs from cDNA. In subsequent cycles, this antisense RNA and cDNA act as a template, and the cycle continue. Eventually, the accumulation of antisense RNA is created that is exactly like the original RNA. NASBA’s performance is far superior to that of RT-PCR, as NASBA can produce up to 10^9^ times the RT-PCR of the product in 1.5 to 2 hours at 41°C.^96^ Since the NASBA products are ssRNA; the denaturation step is omitted to detect hybridization, thus increasing the speed and ease of operations (Figure 5).

The use of NASBA is extended to the rapid detection of bloodstream infections (BSI).^97^ BSI is one of the leading causes of death in the US.^98,99^ On the other hand, the inability of a rapid diagnosis fails to control the infection. Although various microorganisms can play a role in causing the disease, including Gram-positive bacteria and Gram-negative bacteria and various fungi, in most cases, BSI is associated with coagulase-negative *Staphylococci* (CoNS), *S. aureus*, *Enterococcus* spp., *Candida* species, and the Gram-negative bacilli including *E. coli* and *Klebsiella* spp.^100^ The low sensitivity and time consuming of blood culture in BSI diagnosis have replaced the traditional culture-based techniques with newer methods such as NASBA. Also, interpreting the results of the identification of antibiotic-resistant bacteria in conventional methods is a time-consuming process.^101^

Another method used for this purpose is real-time PCR, though the complexity and high cost limit its use. However, the accuracy of NASBA detection for blood pathogens and, at the same time, the need for less time to achieve optimal results have made NSABA a popular method. NASBA enabled identifying a wide range of microorganisms and is applied to detect specific genes on 16S rRNA bacteria and 28S rRNA fungi.^102,103^

### Transcription mediated amplification

**Figure 6:**
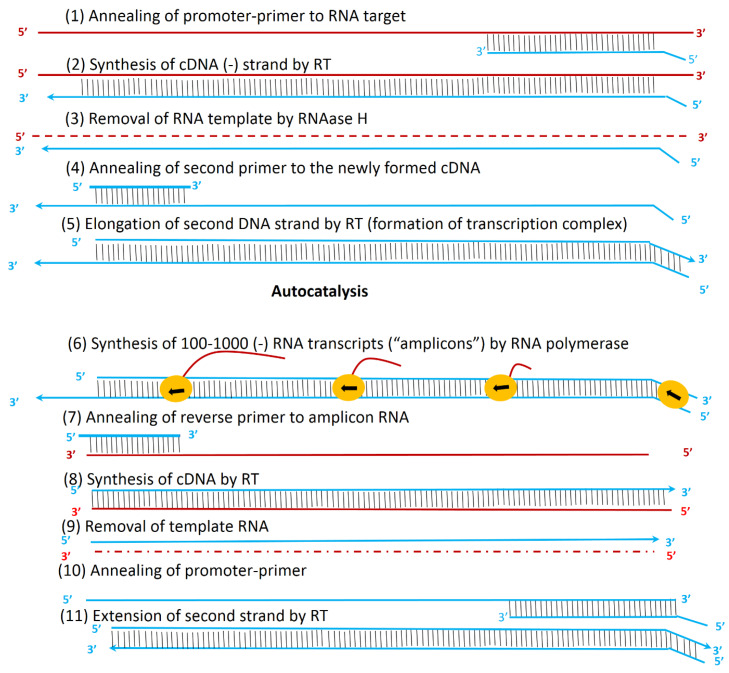
Principle of TMA for the amplification of nucleic acids. Reprinted from Langabeer et al^106^ with permission from Springer Nature Copyright 2002.

As another isothermal amplification system, TMA also works without changing the temperature and requires both polymerase and reverse transcriptase enzymes to amplify nucleic acid. Rapid replication of the target RNA or DNA allows the simultaneous identification of different pathogenic organisms in a single tube (Figure 6). TMA technology enables clinical laboratories to perform a nucleic acid assay for blood screening with shorter process times and faster results.^104^ This method is used in molecular biology and molecular medicine to identify and diagnose pathogenic organisms quickly. Contrary to similar techniques such as PCR and ligase chain reaction, this method for RNA transcription through RNA polymerase and DNA synthesis by reverse transcriptase can create a source RNA product or replication product from a target nucleic acid.^105^ So, this method can be used to target both RNA and DNA. It has many advantages over other amplification methods: (i) it is isothermal; a heated block or a hot water bath is used instead of thermal cycler; (ii) TMA produces RNA replicons instead of DNA. Since RNA is more unstable in the laboratory environment than DNA, this advantage reduces the likelihood of excessive contamination, and (iii) TMA generates 100 to 1000 copies per cycle. This advantage results in an increased number of DNA/RNA copies by 10 billion times in about 15 to 30 minutes.^106^

### Rolling circle amplification

RCA involves two types of hyperbranched rolling circle amplification (HRCA) and primer generation-rolling circle amplification (PG-RCA), which is collectively called exponential RCA.^107^

**Figure 7:**
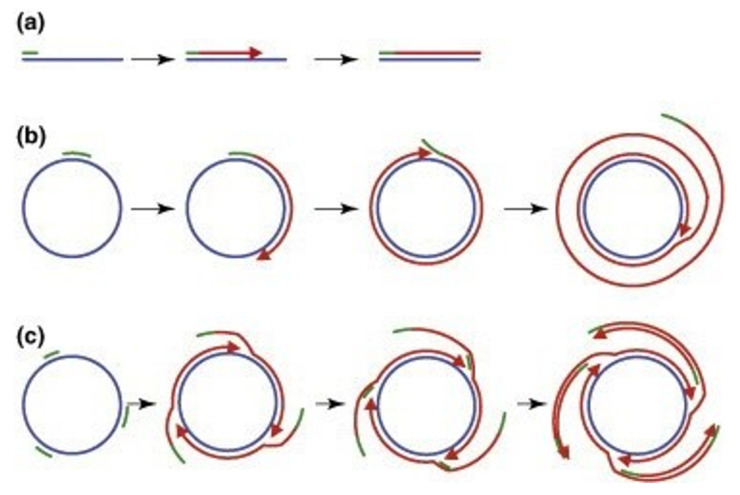
Rolling-circle amplification of viral DNA genomes using Phi29 polymerase. Blue lines represent target DNA, green and red bars indicate oligonucleotide primers and newly synthesized DNA, respectively. Arrowheads demonstrate 3'ends of the growing DNA. **(a)** Primer binds to the template and a complementary strand is synthesized. **(b)** Single primer binds to the circular template and the new complementary strand is synthesized. After completion of one round, the synthesized strand and primer are displaced and a new round starts. The result is the production of several concatemeric ssDNA. **(c)** Multiple random primers bind to the circular template and DNA synthesis occurs in the same way as in b. The multiple products of this reaction are long concatemeric molecules of dsDNA. Reprinted from Johne et al^120^ with permission from Elsevier Copyright 2009.

RCA employs a padlock as a circular template synthesized from ssDNA and is responsible for producing a long and repetitive ssDNA.^108-110^ Two types of enzymes are used in HRCA, including DNA polymerase for the extension of the sequence and DNA-ligase for the padlock probe’s circulation. The primers of this method are of two types; the first primer (P1) is complementary to the padlock probe while the second primer (P2) is paired with the ssDNA made from the padlock probe (Figure 7).^111^

This method’s basis is the strand replacement process leading to the continuous extension and eventually the creation of a segregated series of similar dsDNA fragments. Unlike HRCA, primer generation–rolling circle amplification (PG–RCA) employs nicking sites on the circular template instead of external primers. The nicking endonuclease (NEase) used in this reaction constantly generates these nicking sites to provide a 3́-OH end for DNA polymerase extension activity. The products of both HRCA and PG-RCA methods can be detected by gel electrophoresis and fluorescent dye-based assay.^111,112^

In another study, RCA was used to identify the *rpo*B gene mutation in *Mycobacterium tuberculosis.*^113^ The *rpo*B gene is responsible for encoding the β subunit of the bacterial RNA polymerase that contains the RIF resistance domain (RRDR).^114,115^ Mutations in this gene cause multidrug-resistant strains.^116^ Most RIF-resistant strains are also isoniazid-resistant bacteria. In this study, six lock-in probes were used to detect 12 common mutations in drug resistance in the RRDR *rpo*B gene from *M. tuberculosis.*^117^

As another format of RCA, nick nuclease-mediated exponential RCA is applied for the fabrication of ultrasensitive biosensors. Taken as an example, in 2013, Du et al combined the RCA method with a terminal deoxynucleotidyl transferase (TdT) function. In this method, to improve the technique’s efficiency, the authors used DNase I to produce more -OH ends for the TdT function. During this method, RCA templates were prepared through the generation of dumbbell-like structures by the ligation of two hairpin DNA structures using a T4 ligase activity. The two loops of this dumbbell-like structure contained poly-T sequences acting as primer binding sites. Each loop contained a C-rich sequence to facilitate and improve the recognition of the RCA product. Consequently, the G-rich complementary sequences in RCA products can be folded to create a DNA secondary structure known as G-quadruplexes. Since the thioflavin T shows a precise response to G-quadruplexes, secondary structures play a key role in the detection step, and the RCA process can be tracked using a real-time PCR set.^118^ Likewise, in a study by Jiang et al, authors developed nicking endonuclease-mediated exponential RCA for real-time monitoring of RCA. To this, they employed the specific fluorescence response of thioflavin T to G-quadruplex structures made by RCA products.^119^

### Strand displacement amplification

**Figure 8:**
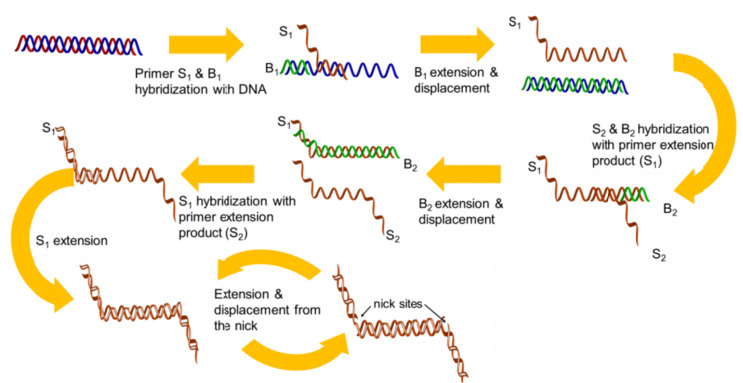
Overview of SDA. Adopted from Chang et al.^123^

SDA technique introduced in 1992^121^ is a DNA replication in which the transcription replication phase is omitted. As a rapid procedure, this isothermal method can produce about 10^7^-fold amplification in 2 hours at 37°C.^122^ SDA employs two types of enzymes: an endonuclease to create the specific 5’ and 3’ ends at the nicking site, and an exonuclease-deficient Klenow to extend the 3’ end. Following heating and denaturation by binding primers P1 and P2, two primer duplication patterns are created that give the 3’ end of the duplexes to expand polymerase and produce dsDNA containing nicking sites. These nicking sites are damaged by the endonuclease (NEase) tip. Following nicking, the newly generated 3’ ends at the nick sites initiate a new extension reaction with the displacement of downstream strand and then the generation of a new nicking site/reaction. The result of the nicking cycles and constant polymerization leads to the production of the target’s ss-compliment. The reaction employs the ss-complements from P1-T1 as the template for P2, while the ss-complements produced by P2-T2 act as the template for P1 (Figure 8).^122^

The continuous repetition of these steps leads to the exponential accumulation of the target DNA. Today, *S. aureus* methicillin-resistant is a model for AMR determination using a novel gene insertion.^124^ Regarding SDA’s specificity, an anchored system is reported for detecting methicillin resistance in *S. aureus.* In one study, the authors purified genomic DNA from cultured methicillin-resistant and sensitive *S. aureus* species and hybridized their DNA to the microelectronic chip array. Positive signals were only reported for MRSA isolates, where the human factor V gene was used as the control in the anchored SDA system. These results proved the specificity and accuracy of anchored SDA performance in methicillin-sensitive amplification reactions.^125,126^

The advantages and disadvantages of above discussed methods are listed in Table 1.

**Table 1:** Comparison of isothermal methods for amplification of nucleic acids

**Isothermal method**	**Tm** (◦C)	**Time**	**Yield**	**Amplicon**	**Requirements**	**Primers**	**Template/ sensitivity**	**Ref.**
LAMP	60 -65	15-60 min	10^9^-10^10^	Various amplicons in size (300 bp -10 kb)	DNA Pol (Bst)	3 pairs	DNA/RNA	^82,89^
RPA	37 -42	5-7min		100-250	Recombinase (T4 UvsX), strand displacing DNA Pol (POL I, Bsu)	1 pair	DNA and RNA	^36-38,127^
RCA	25–37	1h	10^7^-fold	Long ssDNA	DNA Pol (Bst, ϕ29, vent (exo-), T7 RNA Pol)/ nicking endonuclease (NEase)	1 single primer and padlock probe	circular ssDNA, RNA / microRNA	^111,127^
NASBA	41	1.5h	10^9^-10^12^	RNA products	DNA-dependent RNA pol (T7 RNA Pol), RNase H, and (RT avian myeloblastosis virus (AMV) )	1 pair	ssRNA, tmRNA, rRNA	^97,128^
HDA	60-65	1-1.5h	>10^6^	<150bp	thermostable helicase (Tte-UvrD)/ DNA Pol (Bst) or RT	1 pair	DNA, rRNA	^77,81,129^
SDA	37	2h	10^7^	-	Exo- klenow, nicking endonucleases* (Hinc*II)	2 pair	DNA	^122,130^
MDA	30	2h	-	Producs larger than PCR ( > 70 kb)	high processivity DNA Pol (ϕ29)	Random hexamer	dsDNA	^71,131^
TMA	40-55	15-30 min	10^12^	RNA products	RNA polymerase (T7), *RNase H*activity of theRT	2 primers	rRNA	^104,106^

Abbreviations: polymerase (Pol); reverse transcriptase (RT); Endonuclease exonuclease-deficient klenow (exo-klenow).

## Isothermal amplification methods integrated with nanotechnology and microfluidics

This section discusses the combination of isothermal amplification methods with two current advanced systems, namely nanoplatforms and lab-on-a-chip (LOC) microfluidic platforms.

***Combination of isothermal methods with nanoplatforms (ultrasensitive biosensors*** *)*

Nanoscale molecules with high potential to compute at a nano-sized level became much attractive to the researchers.^132^ This is due to the special characteristic of DNA and RNA in recognition and hybridization to a complementary sequence, making them useful biomaterials to build the molecular logic gates.^133^ DNA amplification methods, including PCR-based techniques and thermal procedures, are widely used in clinical applications, especially diagnostic approaches.^134^ The specificity and sensitivity of these methods are significant. The exponential amplification of DNA/RNA as a core technology in modern clinical diagnostics is used in constructing DNA sensors, DNA nano-machines, and DNA sequencing.^107,135^

With regards to the development of supersensitive POC devices, the unique spectral features of nanomaterials such as gold (Au NPs) nanoparticles, called surface plasmon resonance (SPR) can be exploited for signal amplification in the fabrication of optical biosensors in which the detection systems are based on recognition of specific DNA (RNA) target.^127^ Further isothermal amplification of captured (target) nucleic acids and then signal amplification using inherent features of Au nanoparticles (SPR effect) as well as other (metallic and non-metallic (organic)) nanoparticles (metallic nanoparticles) realize a specific yet ultrasensitive DNA-based biosensing system for AMR detection.^136^ Another merit is the regeneration of the sensor surface that permits repetitive uses (at least five cycles) with a shortened assay time.^137^ In this line, LAMP combined with the SPR-DNA arrays is shown to increases the sensitivity and specificity of the method in the detection of MRSA. LAMP amplification was conducted from blood cocultures and sputum for 0.23 kbp and 0.18 kbp DNA fragments of *mec*A and *fem*B genes, respectively. The detection limit of LAMP-SPR sensing was ten copies/µl with a good selectivity toward the MRSA.^137^ Equally, rapid detection of the Panton-Valentine leukocidin (PVL) toxin of MRSA exploiting Au NPs in two hours with LOD of ~200 copies of genomic DNA is obtained.^138^

Recently, LAMP combined with nanogold probe (AuNP) is reported to achieve detection sensitivity even below the food safety control (<100 CFU/g for *S. aureus* enterotoxin A gene (sea) detection). In this formulation, LAMP-AuNP could detect as low as 9.7 fg (3.2 sea copies), which were lower than PCR (97 fg or 32 sea copies). Using crude DNA lysates in food samples, LAMP-AuNP detected down to 10 CFU/g for milk and ground pork samples in 27 minutes achieving 97.5–100% accuracy, 97–100% specificity, and 100% sensitivity, superior to the culture method, and comparable to PCR but without a need for a thermal cycler. The culture method also detected 104 CFU/g and 10 CFU/mL for both samples, respectively in 5–7 days.^139^

In one study, Li et al^140^ designed a logic gate in combination with isothermal amplification of DNA based on the nicking endonuclease method. They improved a series of three-input logic gates. Unlike previously developed methods by Chen et al,^141^ which was based on forming interaction, this method was combined with a cycle amplification. Therefore, it could amplify the sample’s low concentrations, leading to an improved detection limit to 100-folds. Another study performed by Xue et al^142^ reported the detection of ultra-low concentrations of DNA targets. In this preparation, the authors developed a system by the combination of magnetic nanoparticles and exonuclease III (ExoIII)-induced cascade two-stage isothermal amplification. For the integration of target binding and signal transduction sequences, a capture hairpin probe was assembled on magnetic nanoparticles. After sensing the analyte nucleic acid, the hairpin probe could be opened and removed by the ExoIII performance, leading to the release of target DNA and the generation of free signal transduction sequence of capture hairpin probe for the continuation of the next circular reaction. The new DNA could be hybridized with a hairpin DNA probe with a partially caged G-quadruplex sequence inside and forms a duplex structure releasing an active G-quadruplex structure. Then the formed duplex domain is digested by the ExoIII, which leads to the recycling of the process and generation of numerous ZnPPIX/G-quadruplex supermolecular complexes. These products were detected by zinc (II)-protoporphyrin IX (ZnPPIX), which acted as the optical label for fluorescent detection. Accordingly, this platform provided a detection limit of 0.75 fM to distinguish mismatch DNA from perfectly matched target DNA, which offers great potential for the early detection and diagnosis of genetic disorders.^142^

**Figure 9:**
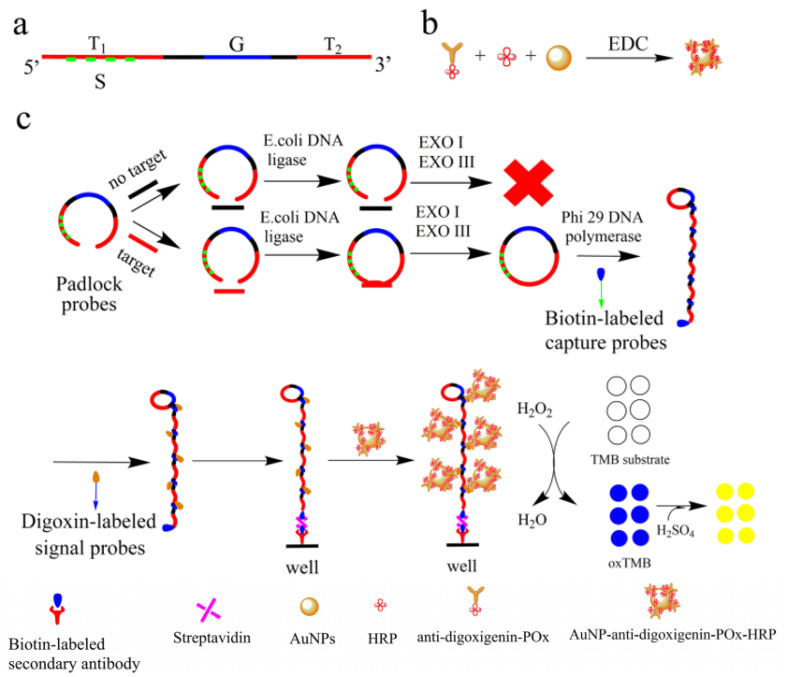
Isothermal methods coupled with nanoplatforms. RCA amplification combined with gold NPs is used for signal amplification to detect AMR with ultra-sensitivity. Reprinted from Li et al.^112^ with permission from Springer Nature Copyright 2020.

Later, Li et al^143^ used a similar method to detect bis-phenol A (BPA) in drinking water and food packaging materials. They developed a label-free aptamer fluorescent assay based on RCA/ExoIII-combined cascade amplification program. BPA recognition and signal amplification required the duplex DNA probe design containing anti-BPA aptamer and the trigger sequence. Then, in BSA’s presence, the trigger probe can be released and initiates RCA reaction to start the primary amplification. The RCA products work as the initiator of the secondary amplification assisted by ExoIII accompanied by hairpin probes to generate a large amount of G-quadruplex in lantern-like structures. Lastly, the G-quadruplex lanterns were illuminated by ZNPPIX to detect fluorescent signals. This method’s most important advantages were excellent sensitivity with a detection limit of 5.4×10-^17^M and high specificity in detecting BPA. In a recent study, RCA was used to determine the concentration of *S. aureus* by colorimetric determination. In this method, linear padlock probes were used to track the target sequence so that these probes, after binding to DNA, were labeled with biotin and act as a primer for RCA. As a result, RCA products containing biotin labels were hybridized with digoxin-labeled signal probes, which were fixed on a plate. The change in color from colorless to blue due to the oxidation of tetramethylbenzidine by H_2_O_2_ made it possible to track the bacteria, resulting in 1.2 pM LOD for the target sequence of *S. aureus* (Figure 9).^112^

In another study, Sedighi et al^144^ integrated the HDA method with gold nano-particles to improve the helicase enzyme denaturation efficiency. This method’s advantages were improved denaturation, enhanced sensitivity, and specificity, and inhibited primer-dimer formation. Likewise, in an effort, Lin et al^145^ conducted a digital LAMP or RT-LAMP on a commercially available membrane to eliminate the intricate chip fabrication need. The procedure involves the effective droplet formation on a commercial track-etched polycarbonate membrane, wherein each single DNA amplification reaction occurs in a pore which works as a nano-reactor. Authors were able to perform absolute quantification of bacterial genomic DNA with a dynamic range of 11-1.1×10^5^ copies/µL. This method was also used for the quantification of MS2 bacteriophage in wastewater. The discrimination between positive and negative probes can be done easily with a 100 times difference in fluorescence intensities. This method can be applied as a low-cost, flexible, and simplified system for POC users or common laboratories.

In an additional setting, Khater et al^146^ developed a label-free highly integrated with *in situ* RPA amplification and detection system needing room temperature to perform the reaction. This method uses the advantages of AuNPs-modified sensing substrates and electrochemical impedance spectroscopic detection. As the proof-of-concept, *Citrus tristeza* virus was detected by this system. The authors transferred the optimized RPA conditions to the AuNP-modified electrode surface previously modified with a thiolated forward primer. The *Citrus tristeza* virus target was amplified *in situ* and analyzed by EUS in a Fe (CN_6_)^4^-/Fe (CN_6_)^3^- red-ox system. This system is potentially able to detect 1000 fg/µL of nucleic acid. The lower cost, higher sensitivity, and portability made this system superior to the RPA in a homogeneous phase.

Notably, isothermal-based amplification methods combined with nanoplatforms are commercially available as test kits for the fast detection of pathogenic agents. In 2019, Thongphueak et al^147^ developed a rapid test kit to identify *Campylobacter spp.*in meat products using LAMP combined with lateral flow dipstick (LFD) and AuNPs. To this, they designed LAMP primers using the conversed nucleic acid region of *Campylobacter spp.* The sensitivity of the LAMP-LFD and LAMP-AuNP assays were measured as 360 fg/µL. The specificity of the analysis was high due to the absence of any cross-reactions to *L. monocytogenes, S. typhimurium, E. coli, B. cereus, P. aeruginosa, S. aureus, E. aerogenes, S. marcescens, V. parahaemolyticus, V. cholera, K. oxytoca,*and* C. diversus*. This POC showed a sensitivity of 100%, specificity of 95%, and an accuracy of 96.67% for both LAMP-LFD and LAMP-AuNPs.

### Combination of isothermal amplification methods with microfluidic devices (high-throughput screening arrays)

Medical diagnostics, environmental observations, and quality control of food products require nucleic acid replication techniques such as PCR. High-efficiency multiplexing techniques have been identified as advantageous in micro-formulation, especially in single-drop microfluidics. The fundamental principle of separating the analyzed sample into a number of identical micro volumes gives the possibility of individual manipulation.^148^ Each analog droplet is isolated from the reaction chamber, which has all the reaction’s necessary components. Simultaneous reacting of thousands of drops results in the development of various new applications that were not identified using other techniques. That is, digital replication methods provide more sensitive and reliable measurements of nucleic acid values.^149^ And are used for studying point mutations, copy number variations, and clonal replication in next-generation sequencing,^150^ and antimicrobial susceptibility testing (AST).^151^ As one, by incorporating an adaptable microfluidic design, a phenotypic AST system determines the of bacteria existence, classifies major classes of bacteria, detects polymicrobial samples, and identifies antimicrobial susceptibility directly from clinical samples (urine, blood cultures, and whole blood). It can also analyze polymicrobial samples at the single-cell level in 30 minutes. Through a pilot study of 25 clinical urine samples, this system demonstrated a sensitivity of 100% and specificity of 83.33% for pathogen classification and achieved 100% concordance for AST.^151^

Furthermore, microfluidic devices provide the possibility of using a tiny system to meet the need for complicated and expensive thermal cycler devices to amplify nucleic acids.^152^ Special PCR is one of these alternatives that involve microchannels contained different regions with fixed temperatures.^153,154^ For the first time in 1998, Kopp et al^155^ amplified a 176 bp fragment of the DNA gyrase gene of *N. gonorrhoeae* using this method. Further introduction of oscillating PCR simplified the digital microfluidics, as this method integrates the speed of the continuous flow PCR with the cycling flexibility of stationery chamber-based PCR.^156^

The marriage of microfluidic devices with isothermal amplification methods to realize effective AMR biosensing platforms has been quite fruitful. With this in mind, and arrayed emulsion droplet microfluidic device has been used for digital LAMP analysis. In the sense of isothermal amplification, a microfluidic droplet array chip can be designed to execute the digital LAMP process by creating emulsion droplets, sorting them into a 30 × 8 droplet array, and executing LAMP across the 240 trapped and separated droplets (with a volume of 0.22 nL) after only 40 min of reaction at 56 °C. This design reported accurate quantification of nucleic acids across a dynamic range of 50 to 2.5 × 10^3^ DNA copies per μL, with LOD down to a single DNA molecule.^157^ For one, Tupik et al^158^ designed the study based on microfluidic mixing with LAMP technique by soft lithography. Authors developed microfluidic devices for fluorescent detection of isothermal propagation of target DNA in a droplet-based microfluidic format as new digital amplification methods with a more reliable and sensitive measurement of nucleic acids. In another study, Lutz et al^159^ developed an RPM microfluidic system to identify the *mec*A antibiotic resistance gene of *S. aureus* for the identification of less than a copy of this gene in <20 minutes.

**Figure 10:**
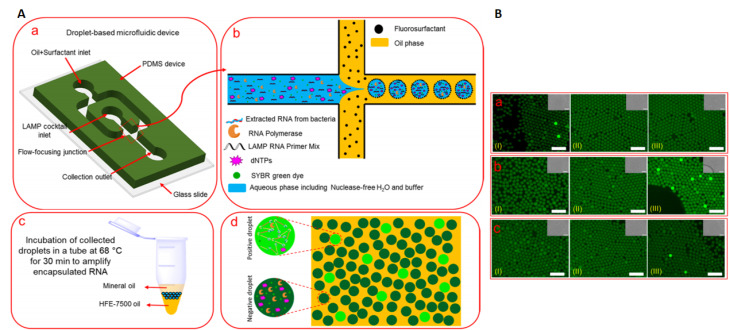
LAMP-assisted droplet microfluidics. **(A)** Schematic representations of sample encapsulation, incubation, and screening. a, the LAMP reaction cocktail is encapsulated into water-in-oil droplets in the microfluidic flow-focusing device. b, Schematic cross-junction view of the flow-focusing, showing Collection of water-in-oil droplets into a PCR tube. c, Incubation of collected droplets (68°C for 30 min). d, Fluorescent imaging and "Yes/No" quantification of the incubated positive droplets. **(B)** Specificity of the LAMP-assisted droplet-based microfluidics sensor to detect the targeted bacteria. Reprinted from Azizi et al^160^ with permission from American Chemical Society Copyright 2019.

Moreover, a combination of microfluidics with nucleic acid amplifications brings about an unprecedented opportunity to fabricate high throughput analytic systems saving both reagent, time, and above that can work for low numbers of RNA/DNA templates. Owing to high specificity and sensitivity, isothermal-based microfluidics systems are very beneficial for diagnostic applications at the time of bacterial or viral outbreaks. Such that, droplet-based microfluidics combined with LAMP is used as a sensitive biosensor for amplification of extracted RNA templates of the *inv*A gene to detect *S. typhimurium* by simultaneously performing ∼10^6^ LAMP-assisted amplification reactions in picoliter-sized droplets. Then, incubation of collected droplets in a thermocycler at 68 °C for 30 min and subsequent fluorescence emission for positive droplets were quantified. This system’s advantages were its simplicity in operation, high sensitivity (1 positive droplet per 250 CFU of *S. typhimurium*), specificity, rapidity, and above all, the high-throughput nature compared to well-established conventional methods. LOD for detecting pure *S. typhimurium* was 5000 CFU/mL in the sample or 25 RNA template/25 μL LAMP reaction cocktail compared to LOD of 5 × 10^5^ CFU/mL of bacteria in the milk sample. This method showed remarkable higher specificity in distinguishing *S. typhimorium inv*(A) gene from two other prevalent bacterial contaminating milk, including *S. flexneri* and *S. aureus* (Figure 10).^160^

Moreover, NASBA on a chip was demonstrated in 2004 by Gulliksen et al^161^ by the real-time detection of a 1.0 mM human papillomavirus virus (HPV) in volumes as low as 10 nL in 41°C silicon-glass microchambers. These results were well compared to conventional NASBA with only a ratio of 1 to 2000 volumes of reaction and demonstrated the first records for the use of NASBA is a miniature identification system. Likewise, Ramalingam et al^162^ designed a real-time HDA microfluidic device built on a glass-sealed PDMS that contained parallel 5 mL microchambers fed by microchannels from a single pipette loading port. Also, Burns et al^163^ described a nanoliter silicon-glass apparatus to detect DNA using SDA and further electrophoresis at 17 minutes amplification time at 50 °C.

Surface-dependent ARCs were used by Sato et al^164^ to develop a fully incorporated RCA microchip using hybrid padlock probes with 34 mm diameter beads as retainers of oligonucleotides and surfaces for RCA. A microchannel with a width of 210 mm and a depth of 40 mm, which holds a 0.5 ml of volume, was made on a glass with a cantilevered structure to hold the beads in place during the various stages of washing, injection of RCA solutions, and introduction of fluorescent probe for detection. The reaction temperature of 30°C and the performance of RCA microchips were demonstrated by *Salmonella* identification. In another attempt, Pardy et al^165^ introduced a resistive heating alternative for disposable LoCNAAT devices. In this device, the author’s employed four-element samples with the different temperature dependence of resistivity profiles, which were by their favorable 60°C to 63°C temperature range. The temperature of sample BM117-83-B1 (one of the four used samples) reached the target temperature range for 5 minutes and was capable of holding this temperature for 25 minutes.

Moreover, a steady-state simulation analysis demonstrated that 85% of the reaction could keep the target temperature range. Eventually, the fabricated device was assessed using the LAMP assay to detect *Chlamydia trachomatis* template DNA. The same group^166^ used a LoCNAAT method combined with LAMP to design a LOC instrument for isothermal amplification methods. The reaction was performed using a Bsm polymerase enzyme within the 20-25°C reaction temperature range. The device was set to sense *C. trachomatis* during an approximately 32-minute time. The advantages of this device were a smaller size, lower energy consumption, lower cost, and easily disposable.

Later, Pardy et al^167^ provided a more completed device of the recent innovations, which was of low cost and wireless smart thermostat device for isothermal nucleic acid amplification as small as a 2 cm×3 cm LoC cartridge. It was equipped with Bluetooth connectivity, a 1200 mA battery, and a built-in OLED display and buttons for local status monitoring. The device provides 60ºC as the reaction temperature. When the LoC is filled with water, it takes 1.5 minutes for the heater and 6 minutes for the microreactor to reach this temperature (total 7 minutes). This device’s battery life was assessed and estimated a 3 hours, 15 minutes, and 55 seconds lifetime. The device supports isothermal nucleic acid amplification technology protocols with 35°C to 65°C range. The most important advantages of this prototype include full portability, the possibility of remote controlling and monitoring, and a fair price for good device performance.

## Conclusion

Together, isothermal methods provide fast, available, portable, simple, and reliable tests for AMR detection and realize POC diagnostics to control infection, particularly in regions with limited sources/facilities. Furthermore improvements in isothermal amplification methods is achieved by their combination with other methods, such as nanotechnology, LOCs and microfluidics. This can pay the way for the development of rapid, available, ease of use miniature POC devices with high sensitivity, specificity, and multiplexing potential to detect simultaneous infections on a tiny paper strip/plastic chip. However, there are available commercial POCs, taken as an example glucose or blood pressure sensors, POCs for AMR detection are urgently needed and actively pursued.

## Ethical Issues

Not applicable.

## Conflict of interest

The authors declare no conflicts of interest.

## Acknowledgments

This study was granted by the Tabriz University of Medical Sciences (TUMS).
